# Hypodermic Injections of Antipyrine in Painful Diseases

**Published:** 1888-05

**Authors:** S. Fraenkerl


### HYPODERMIC INJECTIONS OF ANTIPYRINE IN PAINFUL DISEASES.

BY S. FRAENKERL, M.D., IN "DEUTSCHE MED. WOCHENSCHRIFT" 41, 1887.

The first experiment with this drug made by F. was in an anæmic girl twenty-two years of age, suffering with subacute rheumatism of her right ankle, which had lasted two weeks and had been treated by a variety of remedies, such as salicylate of soda, salol, iodide of potash, antipyrine internally, etc., but without effect. The swelling in the joint continued to increase, especially during the last three days; the pains on the external surface were so severe that the patient could not rest day or night; neither active nor passive motion was possible, even the least touch caused pain. F. injected 0.5 (7½ grs.) antipyrine with a Pravaz syringe in the place of greatest tenderness, and was astonished to find almost instant relief. The patient could now raise her leg, even strong pressure produced no pain; sleep and appetite returned and her general condition was greatly improved; pulse and temperature were not influenced by this treatment. In the afternoon slight tenderness returned on the inner surface of the ankle, which was relieved in the same way the next morning. The whole region of the joint was now entirely free from pain.

The dose recommended by Germain See to produce analgesia of a part is 0.5 ($7\frac{1}{2}$ grs.) *i. e.*, a Pravaz syringe-full of a 50 per cent. solution (antipyrine and water); but F. found that half a syringe-full, *i. e.*, 0.25 antipyrine will produce the same effect, and that we can, therefore, make three or four injections at the same time, in different localities, without having to fear any toxic symptoms. The circle made anagesic by the injection, measures about six or seven per cent (about $2\frac{1}{2}$–3 in.) in diameter. The injection should be made in the direction of the pains, which can always be ascertained by a careful examination. In ten cases treated by this method F. observed that a dose of 0.25 antipyrine is at least equal in its local effect to an injection of 0.02 (gr. $\frac{1}{3}$) morphine; and that this effect is produced in, at most, ten to fifteen seconds, and lasts much longer than that of morphia. The duration of analgesia was, at least, six to eight hours; in most of the cases the pains returned in a much milder degree or not at all in this locality. This method was also successful in the examination of injured joints, the diagnosis of which would otherwise, perhaps, only have been made under an anæsthetic.

F. tried this also in cases of neuralgia and chronic muscular rheumatism in which it not only had a palliative, but occasionally a permanent effect.

A sailor, forty one years of age, suffered with neuralgia of the sixth intercostal nerve for three weeks, in which the treatment by quinine, iodide of potash, salicylates and bromide of potash failed; two half quantity injections in the mammillary region over the rib had such a marvellous result that the patient, who was not able to follow his occupation since the beginning of this trouble, went to work immediately. The same favorable result was obtained in a case of infra-orbital neuralgia, which had lasted three days, resisting all ordinary medication; half a syringeful sufficed to relieve the patient of her intense suffering, which up to date, three days afterwards, has not returned. He also succeeded in relieving several cases of pleurodynia promptly with one or two injections of half a syringeful.

In all cases of rheumatism affecting the muscles of the neck or loins and in lumbago the hypodermic administration of antipyrine is followed by remarkable results. In the last named affection, F. has always injected it on both sides of the spinal column about three or four c. m. external to it and found that it not only relieved the pains but removed the disease completely, so that no further treatment was necessary except that of a general nature such as rest, etc. In sciatica the method has done good service, especially in the beginning of the affection; two to three injections at the painful points in the direction of the femur have often given complete and permanent relief.

These are the experiences gained by F with this method in the treatment of external painful affections. In internal diseases half, or most, one full injection is sufficient to give relief from pain. In a patient suffering with acute otitis media for three days without rupturing the tympanum, one full injection given in a downward direction gave complete re ief. He was free from pain until the next day, when the membrane was perforated; he is now convalescing. In the same way a patient, twenty-five years old, suffering with the first stage of typhlitis, who had intense pains so that any movement of her body was impossible, was immediately relieved by the injection of a syringeful of the solution in the region of the ileo cæcal valve, so that she could sit up in bed and change her position as desired. A girl, five years old, affected with the same disease, was made easy by the injection of half a dose.

F. is firmly convinced that the sub-cutaneous injection of antipyrine will limit the use of morphia to a considerable extent, and that it will be a great convenience both to physician and patient.—*Pittsburgh Medical Review.*

